# Effect of Broilers Chicken Diet Supplementation with Natural and Acidified Humic Substances on Quality of Produced Breast Meat

**DOI:** 10.3390/ani11041087

**Published:** 2021-04-10

**Authors:** Marek Hudák, Boris Semjon, Dana Marcinčáková, Lukáš Bujňák, Pavel Naď, Beáta Koréneková, Jozef Nagy, Martin Bartkovský, Slavomír Marcinčák

**Affiliations:** 1 Department of Nutrition, Dietetics and Animal Breeding, University of Veterinary Medicine and Pharmacy in Košice, Komenského 73, 041 81 Košice, Slovakia; marek.hudak@student.uvlf.sk (M.H.); lukas.bujnak@uvlf.sk (L.B.); pavel.nad@uvlf.sk (P.N.); 2 Department of Food Hygiene, Technology and Safety, University of Veterinary Medicine and Pharmacy in Košice, Komenského 73, 041 81 Košice, Slovakia; beata.korenekova@uvlf.sk (B.K.); jozef.nagy@uvlf.sk (J.N.); martin.bartkovsky@uvlf.sk (M.B.); slavomir.marcincak@uvlf.sk (S.M.); 3 Department of Pharmacology and Toxicology, University of Veterinary Medicine and Pharmacy in Košice, Komenského 73, 041 81 Košice, Slovakia; dana.marcincakova@uvlf.sk

**Keywords:** poultry, humic substances, nutrition, meat, sensory evaluation, lipid oxidation

## Abstract

**Simple Summary:**

Meat quality can be influenced by incorporating additives into an animal’s diet. Humic substances (HS) are natural products which have the potential to improve the meat quality of broiler chickens. HS are used as antidiarrheal, analgesic, immunostimulatory, and antimicrobial agents in poultry production. The effects of natural and acidified HS supplements on broiler meat quality traits (growth performance, carcass yield, physicochemical composition, lipid oxidation, antioxidant activity of meat extracts, and sensory and colour characteristics) were studied. Both supplements were composed of Leonardite, whereby the acidified HS were treated with formic acid for better digestibility. The breast meat quality of experimental broiler groups fed with HS were affected in total protein and fat content, and both showed lower lipid oxidation and higher antioxidant activity of meat extracts after the storage period (7 days at 4 ± 2 °C).

**Abstract:**

This study was conducted to examine the effect of two humic substances (HS) supplemented in broilers’ diet on the breast meat quality of broiler chickens. In this experiment, 120 pieces of one-day-old COBB500 broiler chickens were used. Broilers were divided into three groups, each containing 40 birds with three replications (13, 13, and 14 per one pen). Fattening lasted 38 days. The first experimental diet was supplemented with 0.7% of HS (HS0.7) and the second was enriched with 0.7% of acidified HS (HSA0.7). The control group of broilers (C) was fed a basal diet without the addition of any supplements. HS0.7 samples had the highest total protein content and the lowest content of fat (*p* < 0.01). The effects of broiler diet and storage had a significant impact on the pH of breast samples, *p* < 0.001 and *p* < 0.05, respectively. Supplementation of HS in broiler diet positively affected the lipid oxidation of breast meat samples, as indicated by the lower malondialdehyde content (*p* < 0.05). HSA0.7 samples had significantly better juiciness after the storage period (*p* < 0.001). The quality of meat produced with supplementation of HS in broilers’ feed can be considered as valuable in human nutrition due to improved protein and fat content with decreased lipid oxidation.

## 1. Introduction

In commercial poultry, the production of broiler feed contributes to up to 70% of the total production cost [1]. It can be concluded that poultry meat production depends on feed as one of the main factors [2]. There are considerable differences in bird response related to the nature of ingredients that are either not used in all diets or are incorporated at different levels [3]. Due to increases in global feed prices, there is a tendency in the poultry industry to move towards alternative or unconventional feed ingredients [1]. Considering cost, taste, and nutritional value, the meat of broilers is one of the most popular meats in human nutrition [4]. Moreover, the Food and Agricultural Organisation stated the importance of chicken meat in human nutrition for its physicochemical quality, because of its high-quality protein content and a low level of fat [5].

Organic acids, including humic acids, have made a great contribution to the profitability in the poultry industry and have provided people with healthy and nutritious poultry products [6]. In the European Union, the search for alternative feed supplements in animal production has been promoted due to the ban on the use of antibiotics as growth promoters [7]. Moreover, birds treated with antibiotics can no longer be marketed as organic and may contribute to antibiotic resistance development via the food chain [8].

Humic substances (HS) are produced by disintegration of organic mass, particularly herbal and soil components [9]. They are able to affect weight gains positively and enhance function of the immune system [10]. Certain modified forms of humic acid have been shown to possess antiviral activities [11]. Vašková et al. [12] also studied the possibility of preventing the consequences of chronic lead poisoning by the administration of three different doses of HS into feed. HS are advantageous as they are non-toxic, non-teratogenic, and withdrawal periods are not needed [6,13]. Therefore, performance and disease control using HS are the most important factors that affect the efficacy of poultry production [14]. As one of the alternative feed additives, humic substances (including humates, humifulvates, humic acids, and fulvic acid) are being currently used in animal husbandry [15].

There are many parameters that can improve the quality of meat during the meat production process [16,17]. Tang et al. [18] concluded that muscle colour and texture are the two most important factors that influence meat quality. Colour, pH, water-holding capacity [13], and visual acceptance [19] are also important characteristics that can affect consumer preferences in chicken meat [20].

Many authors reported the impact of HS in poultry meat production [2,13,14,21,22]. Our previously published studies evaluated the meat quality of broilers, after their diet was supplemented with 0.8 and 1.0% additions of natural HS to the feed mixture pellets [2,14]. Ozturk et al. [13] reported a positive impact on feed conversion and meat quality [13]. The administration of HS in drinking water and the impact on broilers’ growth, carcass yield, and gut characteristics was evaluated by Ozturk et al. [21].

The aim of our experiment was to assess the effect of two different HS supplements in the diet of broilers. The dietary natural HS and HS acidified with formic acid were supplemented in broilers’ feed mixtures and the impact of both HS supplements was studied on the physical and chemical parameters of broilers’ breast meat. To the best of our knowledge, no such information, which compares the application of acidified and non-acidified HS supplements in broilers’ diet, has been available to date. Furthermore, the same status applies to evaluating broilers’ meat quality supported with sensory evaluation and colorimetry.

## 2. Materials and Methods

This study was conducted in accordance with the guidelines of the Ethics Committee for the Use of Animals in Research of the University of Veterinary Medicine and Pharmacy in Košice. All procedures in the present study were performed in accordance with the principles of the European Union and Slovak Law on Animal Protection. The experimental diets were not toxic and according to the regulation 68/2013, from 16th January 2013, the European Union Commission allows the use of Leonardite as a source of humic substances as a feed component in animal diets. Furthermore, the experiment was approved and carried out with the consent of the State Veterinary and Food Administration of the Slovak Republic under the protocol no. 3040-14-221.

### 2.1. Birds, Housing, and Feeding

Two different humic substance supplements (Humac, Ltd., Košice, Slovakia) were used in this experiment. Both were composed of Leonardite, a naturally occurring mineral complex consisting of phenolic hydrocarbons, also known as humate, which comes from the decomposition of organic material that occurs over a period of millions of years. It consists of humic acids, free humic acids, fulvic acids, and minerals. The dietary natural HS supplement was grounded and physically purified Leonardite without chemical treatment. It contained natural humic substances with more than 65% of humic acids, without acid salts. The second HS supplement was grounded, physically purified Leonardite acidified with formic acid for the purpose of increasing the digestibility in monogastric animals. The composition of HS supplements is presented in Table 1.

Feeding of broiler chickens was divided into the three fattening periods, which lasted a total of 38 days during this experiment. The broiler chicken feed mixtures were obtained from DeHeus (Bučovice, Czech Republic) and were fed to the control and experimental groups. These feed mixtures were fed according to producer recommendations. Each feed set contained the same amount of metabolisable energy and crude protein. The main components of broiler chicken feed mixtures were wheat, corn, soybean meal, rapeseed cake, and sunflower meal (Table 2). At the beginning of fattening, feed mixture BR1 (starter diet) was administered during the first 10 days of fattening. From day 11 to day 28, birds consumed growing diet 1 (BR2). From day 29 to day 38 birds were fed final diet (BR3). Chickens of the experimental groups were fed the same, but HS were administered during each of the three fattening periods. For administration of HS in experimental diets, the whole batch of individual experimental feed mixtures was prepared 24 h before each feeding phase began. The finely ground diets were prepared by a bucket electric grain grinder, which was driven by a 1.2 kW single-phase electric motor, equipped with 1500 μm sift (AMA S.p.A., Reggio Emilia, Italy). Subsequently, the fine mash of each ground basal diet (max. particle size 1500 μm) was well mixed with each HS supplement (max. particle size 1500 μm) by 145 l compact mixer (Altrad Group, Montpellier, France) for 15 min. Thus, a consistent dispersion of feed and HS particles was achieved.

For this experiment, 120 pieces of male broiler chickens COBB 500 (*Gallus gallus domesticus*) from farm Mach Hydina Budmerice Ltd. (Budmerice, Slovak Republic) were employed. Broilers were divided into 3 groups, which were differentiated by the experimental dietary treatments. One group consisted of 40 pieces, with 3 replications (13, 13, and 14 birds per one pen). The control group (C) was fed with a feed mixture without supplementation of HS during fattening. Two experimental groups were fed with feed mixture, one supplemented with 0.7% dietary natural HS (HS0.7), and the second with 0.7% acidified HS (HSA0.7).

The fattening of the chickens was carried out on deep litter. Conditions (light, temperature, and humidity) in the breeding facilities were controlled constantly [23]. There was a 24-h light regime for the first day, which was declined to an 18-h regimen during the first week. Temperature was set at 33 °C on the first day and gradually dropped to 21 °C until the 24th day. Humidity was also monitored and maintained at about 70%. Access to water and feed was ad libitum.

### 2.2. Data Collection and Chemical Analyses

On day 38 of the trial, after 12 h without feed, chickens were individually weighed, euthanised by cervical dislocation and bled out. To investigate the growth-promoting effects of natural and acidified HS on broiler performance, the overall body weight and feed consumption were recorded, and total weight gain with feed conversion ration were calculated. The carcass weight was recorded after slaughtering (decapitation, hock cut off and evisceration). The yield of carcass was determined as a ratio of the final body and carcass weight. The breast muscles, thigh, wings, and hulls were weighed and their percentage values were calculated.

The breast meat (musculus pectoralis major) samples were stored at 4 ± 2 °C until meat quality analysis. For the analysis of meat quality, 18 broilers of each experimental group were randomly selected (i.e., six birds per replicate). In these samples, dry matter, water, fat and protein content, pH, malondialdehyde (MDA) content, antioxidant activity (AA) of meat extracts, and sensory characteristics were evaluated. For pH, MDA, AA determination, and sensory assessment of breast meat, samples were stored for the next seven days, and then used in analyses.

Content of dry matter was determined by oven-drying at 105 °C [24], using a Universal Oven UN 110 (Memmert GmbH + Co. KG, Büchenbach, Germany), and crude protein content was determined by a Kjeltec auto type 1030 analyser (Tecator Co., Hoganas, Sweden). Lipids were isolated in samples with petroleum ether in Soxhlet apparatus (LTHS 500, Brnenská Druteva v.d., Brno, Czech Republic) and were determined gravimetrically. The pH of meat samples was analysed with a digital inoLab^®^ pH 340i meter (Wissenschaftlich-Technische Werkstätten, Germany). To determine the lipid oxidation changes of breast muscles, the 2-thiobarbituric acid spectrophotometric method was used. The extent of lipid oxidation involved the measurement of thiobarbituric acid reactive substances (TBARS), as prescribed by the method of Reitznerová et al. [25]. TBARS values were measured spectrophotometrically at 532 nm (Helios α, v.4.6 Thermo Spectronic, Cambridge, UK) within 24 h after slaughter and after 7-day storage in a refrigerator (at 4 ± 2 °C). Results were quantified as MDA equivalents and expressed as mg of MDA/kg of sample. Antioxidant activity of breast meat samples was measured spectrophotometrically by the method of 2,2-diphenyl-1-picrylhydrazyl (DPPH) scavenging activity [26]. The DPPH solution in methanol (0.1 μM, 0.0025 g/100 mL) was prepared before analysis. The prepared DPPH solution (3.8 mL) was placed into a 1-cm thick cuvette and the extinction was recorded at 515 nm. Subsequently, 200 μL of the prepared breast meat extract was added into the cuvette and mixed. The cuvette was left for 10 min at laboratory temperature to react and then, the absorbance was measured. The DPPH radical-scavenging capacity of breast meat extracts was calculated and expressed as % of DPPH radical inhibition.

The colour of meat samples was quantitatively measured by a Chroma meter CR-410 (Konica Minolta, Sensing, Inc., Osaka, Japan) using Colour Data Software CM-S100w SpectraMagic NX (Konica Minolta Sensing Inc., Osaka, Japan). The colorimetric data were obtained using the following device set up: measurement area ∅ 50 mm, illuminance D65, and standard observer angle 2°. The Chroma meter was calibrated throughout the study using a white standard plate (CR-A43, Konica Minolta, Sensing, Inc., Osaka, Japan). Colour parameters were expressed in Lab and LCh colorimetric space according to the International Commission on Illumination values (CIE) and McLaren [27]. The L* value represents lightness, a* value represents redness (chromaticity from green (−a) to red (+a)), and b* value represents yellowness (chromaticity from blue (−b) to yellow (+b)). The hue angle (h*) and chroma (C*) parameters were expressed from the combination of a* and b* according to McLaren [27]. The measurements were performed in a laboratory room at 20 ± 2 °C. The results reported are average values of the total of 18 measurements.

The sensory assessment was carried out in a standardised sensory laboratory built according to the general guidance for the design of test rooms [28]. The evaluation was performed by 12 trained panelists ranging from 28 to 65 years old. The evaluation was performed on the 1st and the 7th day of storage. The samples were stored under controlled conditions at a temperature of 4 ± 2 °C and without light exposure until the moment of the analysis. The procedure for preparation of breast meat samples included cooking of the meat in boiled water (until a temperature of 80 °C was achieved in the core of the meat) and portioning of cooked meat samples into square cubes (with a weight of approximately 25 g). The samples were coded with random three-digit numbers and a dish with water for mouth-rinsing was provided for evaluators. The overall appearance, aroma, taste, and acceptability of each sample were evaluated using a 9-point hedonic scale from 1 (dislike extremely) to 9 (like extremely). The intensity of breast meat juiciness and brittleness attribute were scored on a structured 10 cm line scale anchored as “not perceptible” at the low end and “intense“ at the high end.

### 2.3. Statistical Analysis

The results obtained in this experiment were expressed as means and the pooled standard error of the mean (SEM). One-Way and Two-Way Analysis of Variance (ANOVA) with Tukey’s and Sidak´s tests for multiple comparisons of means were carried out via the software GraphPad Prism 8.3 (GraphPad Software, San Diego, CA, USA). Multiple factor analysis (MFA) was conducted in R-statistics 4.0.3 software [29] with “FactomineR” [30] and “factoextra” packages [31], according to Pagès [32] and Semjon et al. [33]. The effects of broiler diet supplementation with two different HS supplements (dietary natural HS and acidified HS) and storage period were set as the main factors. The results of the MFA method were visualised by 2 plots: a combined graph of individuals with the two main examined factors, and a correlation circle. A significance level of *p* < 0.05 was set in each applied statistical method prior to data analysis.

## 3. Results

During the fattening period, neither clinical symptoms of disease, nor abnormal mortality, were observed. Means for growth performance variables, carcass yield, deboned breast and thighs, wings, and hulls are presented in Table 3. Each growth performance parameter did not show significant statistical differences among the treatments (*p* > 0.05). The carcass yield of both experimental groups (HS0.7 and HSA0.7) was increased, when compared to C (*p >* 0.05). The control group had a significantly higher average weight of the wings than both experimental groups where HS was administered in broiler diet (*p* < 0.001).

Statistically significant differences were observed in each analysed physicochemical parameter of breast meat samples at a significance level lower than 0.05 (Table 4). The dry matter measured in HSA0.7 was lower than in C and HS0.7 (*p* < 0.05). A similar significant difference was observed in the water content, whereby a significant increase was determined for breast meat samples of HSA0.7 (*p* < 0.05).

The application of HS in broiler diet significantly decreased the fat content (*p* < 0.01). The total protein content of breast meat samples decreased only in group HSA0.7 (*p* < 0.01). The means of the breast fat content of each experimental group differed significantly opposite the C group (*p* < 0.01), but a statistical difference between HS0.7 and HSA0.7 was not observed. The application of 0.7% of modified HS in broiler diet for the HSA0.7 experimental group led to a significant decrease in total protein content in breast meat samples, when compared to C and HS0.7. The effect of supplementation of broiler diet with HS on pH and MDA is shown in Table 5. The decrease in pH of breast meat samples obtained from broilers of experimental groups, where HS were administered, was significant on both the 1st and 7th day of storage of the samples (*p* < 0.001). The storage effect was observed only in HSA0.7, where the pH of the samples was significantly lower after the storage period (*p* < 0.01).

The storage period affected the lipid oxidation of breast meat samples, which resulted in an increased content of MDA in samples measured on the 7th day of storage (Table 5). This increase was significant only in C and HS0.7 (*p* < 0.05). The lowest increase in MDA after storage was observed in HSA0.7 breast meat samples. A difference in effect of HS application in experimental broiler diet on lipid oxidation in breast meat samples was observed between HSA0.7 and C groups (*p* < 0.05). However, this effect in MDA content was significant only between samples analysed on the 7th day of storage (*p* < 0.05).

Table 6 summarises colour values of analysed breast meat samples, which varied with the dietary treatments and during storage.

Responses of each colorimetric parameter to dietary treatments were significant (*p* < 0.001), except for yellowness (*p* > 0.05). The effect of storage period on the colour of samples was observed in the following colorimetric parameters: lightness, yellowness, and hue angle, at a significance level of *p* < 0.001, *p* < 0.01, and *p* < 0.01, respectively. The L* values of breast meat samples from broilers in the HSA0.7 group on the first day of analyses corresponded to samples with a lighter colour, when compared to those obtained and observed for meat samples from the C group (*p* < 0.05). On the 7th day of storage, a significantly lighter colour for both HS0.7 and HSA0.7 samples was observed, than for C samples (*p* < 0.01). The storage period affected the L* colour parameter of both experimental groups of samples (*p* < 0.001). Yellowness and hue angle of measured samples significantly changed only for the C samples after the storage period (*p* < 0.05).

**Figure 1:**
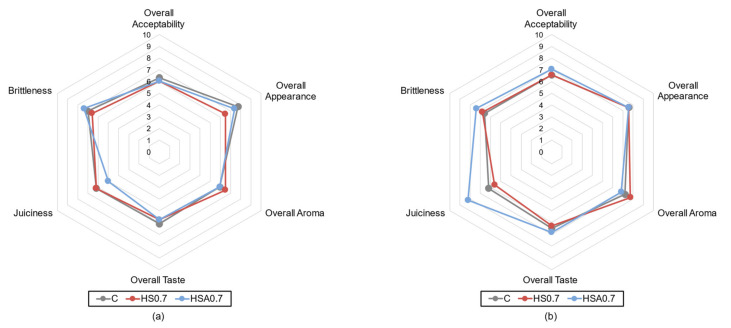
(**a**) The results of sensory evaluation of breast meat samples on the 1st day of storage. (**b**) The results of sensory evaluation of breast meat samples on the 7th day of storage. C: control group; HS0.7: broilers’ diet supplemented with 0.7% of dietary natural HS; and HSA0.7: broilers’ diet supplemented with 0.7% of HS acidified with formic acid.

Sensory evaluation of breast meat samples was performed on the 1st and 7th day of storage at 4 ± 2 °C. The results of sensory evaluation are presented in Figure 1. The overall sensory evaluation of breast meat samples included overall appearance, aroma, taste, and acceptability, which were not affected by the application of dietary natural and acidified HS substances in broilers’ diet (*p* > 0.05). Additionally, the storage period did not change the overall sensory characteristics of the breast meat samples (*p* > 0.05). The quality descriptor of meat brittleness was neither affected by the broiler dietary treatment, nor by the storage of samples (*p* > 0.05). However, the juiciness of the breast meat samples, analysed on the 7th day of storage, showed significant differences between C and HSA0.7, and HS0.7 and HSA0.7 at a significance level of *p* < 0.05 and *p* < 0.01, respectively.

Our obtained results of carcass yield, colorimetric, physicochemical, and sensory analysis of breast meat samples were subjected to MFA statistical analysis, with the application of Kaiser’s criterion (Eigen value > 1) [34] to determine the number of final factors from the initial ones. The results of MFA analysis show that in breast meat samples, four selected components explain more than 62% of the total variation in the data set. The first dimension (Dim1) explains 21.64%, dimension 2 (Dim2) 13.60%, dimension (Dim3) 12.09%, dimension 4 (Dim4) 7.85%, and dimension 5 (Dim5) 7.28%.

The presented results of Dim1 belonged to the physicochemical parameters of breast meat samples (25.98% *r* = 0.91). The correlated parameters in Dim1 included fat (6.62% *r* = 0.74), dry matter (4.09% *r* = 0.64), pH (4.86% *r* = 0.60), malondialdehyde content (3.11% *r* = 0.54), total protein content (0.44% *r* = 0.19), antioxidant activity of meat extracts (2.07% *r* = −0.45), and water content (4.09% *r* = −0.64). Each analysed parameter in Dim1 was correlated at a statistically significant level *p* < 0.001. Dim2 was represented mainly by the sort of meat samples (47.34%, *r* = 0.95).

**Figure 2:**
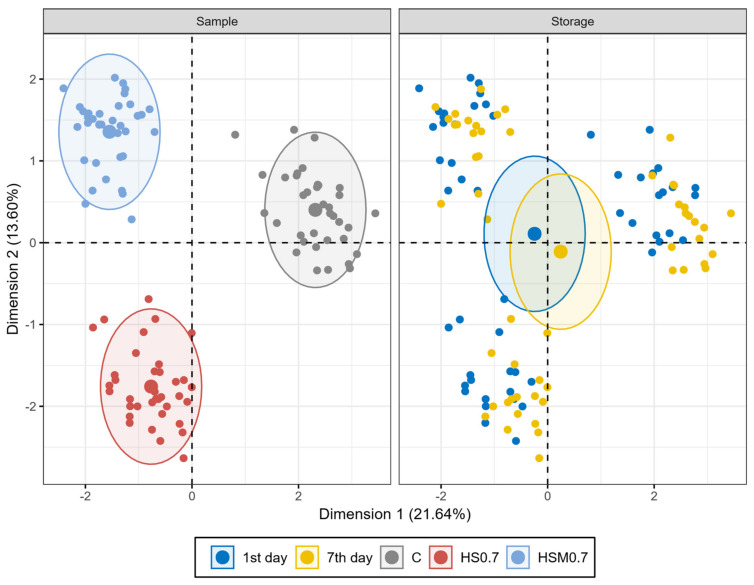
Multiple factor analysis (MFA) plot of breast meat samples: Combined graph of individuals with visualised sample and storage factor. C: control group; HS0.7: broilers’ diet supplemented with 0.7% of dietary natural HS; and HSA0.7: broilers’ diet supplemented with 0.7% of HS acidified with formic acid.

**Figure 3:**
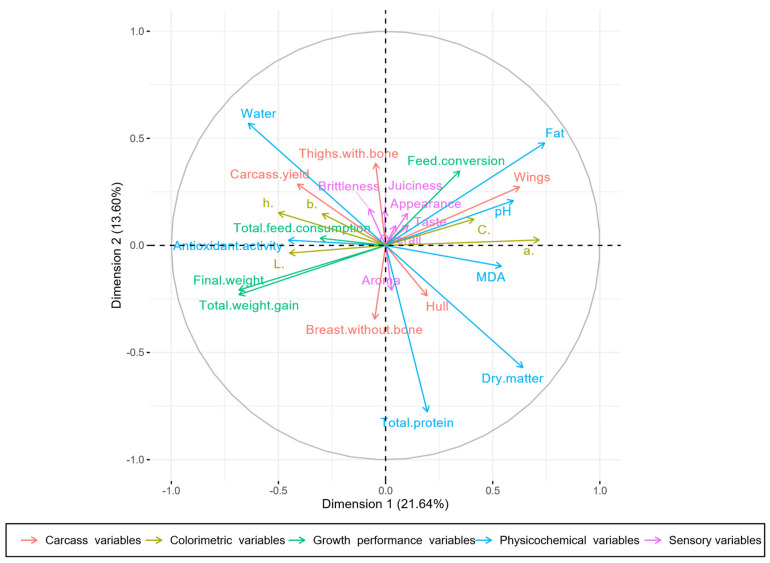
MFA plot of breast meat samples: correlation circle.

A total variance of 35.24% was explained in Dim1 and Dim2 (Figure 2). Analysed physicochemical and sensory parameters of the breast meat samples, which correlated to Dim1 and Dim2, are visualised in Figure 3. Storage effect and sensory characteristics contributed especially to Dim3, with 50.71% (*r* = 0.94) and 16.15% (*r* = 0.54), respectively.

The sensory parameters of breast meat samples correlated at a level of significance of *p* < 0.01 in Dim3: overall acceptability (*r* = 0.51), overall aroma (*r* = 0.46), juiciness (*r* = 0.42), overall taste (*r* = 0.40), and overall appearance (*r* = 0.29). Dim4 was characterised mainly by the carcass yield (44.24%, *r* = 0.73) and colorimetric parameters (32.91%, *r* = 0.61). On characterisation of Dim4, the following significantly contributed and correlated: carcass yield of thighs without bone (9.08%, *r* = 0.38), breast without bone (5.28%. *r* = 0.31), hulls (27.00%, *r* = −0,66), and colorimetric values of b* (13.74%, *r* = 0.62), h* (11.07%, *r* = 0.56), C* (5.18%, *r* = 0.38), and L* (2.34%, *r* = 0.27).

## 4. Discussion

The broiler diet composition showed that broilers of both control and experimental groups were fed a nutrient-balanced diet during each of the three fattening periods (Table 1). When comparing the response of chickens to dietary energy, one of two approaches can be taken: (1) formulation of one diet and then diluting it with materials with little nutritional value or effect, or (2) formulation of diets using practical feed ingredients [3]. The application of HS in HS0.7 and HSA0.7 did not significantly affect the metabolisable energy and crude protein content of the experimental broiler diets, which are important for achieving objective results from such studies.

In our recent published studies [2,14] and in research published by Ozturk et al. [21], a positive effect of HS on feed conversion of poultry and improved quality of produced meat were observed. In contrast, some studies showed a non-significant effect on the broilers during the fattening period [10,35]. Thus, the objective of this study was to observe the effect of supplementing broiler nutrition with either 0.7% dietary natural HS or HS acidified with formic acid. Subsequently, the MFA statistical method was applied on obtained data to evaluate the quality of produced breast meat of broilers fed with natural and acidified HS. Statistical correlations between physicochemical and sensory parameters with colorimetric results were analysed.

According to the obtained results, we can conclude that broiler diets supplemented with HS could have a significant impact on the meat quality characteristics, in which the 0.7% supplementation of natural HS with formic acid tended to be more effective. The supplementation of chicken diet with dietary natural and acidified HS resulted in statistical differences in the following measured parameters of breast meat samples: dry matter, water, fat, total proteins, pH, and MDA (*p* < 0.05). Ozturk et al. [13] reported that the addition of 0.5, 1.0, and 1.5% HS concentrations had different effects on fat and total protein content. A HS addition of 1.0% concentration in broiler diet resulted in decreased total protein content in breast meat samples, while the concentrations of 0.5 and 1.5% did not have a significant effect on total breast muscle proteins. In contrast, the fat content of the breast muscle was slightly lower in the experimental groups than in the control [13].

Lipid oxidation was also affected by the broiler diet modification, and the effect between experimental groups was significant after seven days of storage, when MDA content was significantly lower in the HSA0.7 group (*p* < 0.01). A high oxidative stability of meat is important to avoid or delay development of rancid products or warmed-over flavour. In relation to the character of the process of lipid oxidation, the effect of antioxidants is more significant the earlier they are applied [36]. Marcinčáková et al. [22] reported that after feeding HS of 0.8% to broilers, the meat stored in the refrigerator as well as the meat frozen for 12 months had comparable oxidative stability in comparison to meat obtained from the control group. Another important factor of the good oxidative stability of fats could be the fact that meat obtained after feeding humic substances contained a lower proportion of fat than control meat, as stated in work by Ozturk et al. [13].

Antioxidants are capable of inhibiting the oxidation process, but they are irreversibly consumed in the reaction with peroxide radicals, and they make it possible to slow down the process only for a little while [37].

In general, humic substances are considered to possess components that are able to reduce oxidative stress in the body [38]. Humic substances contain polyphenolic components and show sufficient antioxidant capacity in vitro [39,40]. This effect is probably related to the content of electron-donating phenolic groups. In addition, the presence of acid groups (–COOH, –OH) suggests that these substances are capable of an antioxidant effect [41]. Vašková et al. [42] determined the effects of the activities of antioxidant enzymes and levels of trace element co-factors after a 42-day supplementation of humic acids in normal breeding conditions and under stress conditions caused by transportation of broiler chickens to the slaughterhouse. They found that 0.6% humic acid concentration in broiler diet affected the level of selected enzymes directly involved in oxidative stress elimination [42]. However, there is still a lack of scientific works that have studied the effect of humic components on the antioxidant activity and oxidative stability of meat. In the research published by Domínguez-Negrete et al. [8], the antioxidant status of breast meat was measured to evaluate the influence of HS on broiler breast meat quality, but they did not find a positive impact. On the contrary, our observations of antioxidant activity of meat extracts showed that HS could contribute to higher inhibition of DPPH radical activity in meat samples of experimental groups (HS0.7 and HSA0.7), when compared to the control group.

This could confirm the effectiveness of humic substances as antioxidants in meat fat. The meat obtained after feeding humic substances contained a lower proportion of fat than control meat, as was also stated in the study of Semjon et al. (2020). The lower fat content and the higher proportion of antioxidant components in the meat of the experimental groups could have an effect on the higher oxidative stability. Therefore, further research is needed to verify the effect of HS as a substance capable of increasing the oxidative stability of meat fats.

Food quality could be defined by several terms and factors including food safety, qualitative standards, nutritional values, stability, and main factors for consumers, such as sensory characteristics, which have an important role in consumers’ perception [43]. Meat quality is related directly to stress, and energy metabolism [13,42] and enzymes which affect many aspects of meat quality [44,45,46]. Muscle colour and texture are always two of the most important factors that influence chicken meat quality [2,20]. L* colorimetric value increased in the samples obtained from broilers that were fed a chicken diet administered with HS, and this increase, on the first day of storage, was significant between C and HSA0.7 (*p* < 0.001). Ozturk et al. [13] found that a 1.5% addition of liquid HS in broiler diet during fattening of 42 days resulted in lighter breast meat colour than in groups of chickens where 0.5% and 1.0% of HS were added to broiler diet. On the other hand, our previous study showed a significant decrease in the lightness of breast meat samples with an increase in HS concentration in broiler diet (0.8% and 1.0%, respectively) [2]. After seven days of storage, the lightness of HS0.7 and HSA0.7 samples significantly increased (*p* < 0.001). Our observations showed a significant effect of supplementing broiler diet with HS. In particular, this was true for almost every analysed colorimetric parameter (*p* < 0.001), except the yellowness of samples (*p* > 0.05). According to our results of the instrumental analysis of meat colour, we can conclude that the supplementation of HS in broilers’ diet caused the muscle tissue (musculus pectoralis major) of HS0.7 and HSA0.7 samples to be lighter in colour and the intensity of the redness was lower.

Only a few aspects of breast meat can be perceived by consumers before purchase (e.g., those related to visual aspects, such as colour, marbling, and texture), whereas most of them can be perceived after purchasing, at the moment of consumption [47]. Meat texture belongs to one of the most important sensory qualities associated with consumer´s satisfaction [20] and parameters related to meat texture, such as firmness, tenderness, hardness, and crumbliness are generally determined in most studies related to meat quality [48]. The evaluated factors of broiler diet modification and storage period did not affect the overall sensory evaluation of the samples. The overall appearance, aroma, taste, and acceptability of breast meat samples were not affected by both factors (*p* > 0.05). However, an increasing tendency in the hedonic evaluation of overall acceptance intensities of HSA0.7 and HSA0.7 breast meat samples on the seventh day of storage were observed. Meat brittleness was neither affected by the dietary treatment, nor by storage (*p* > 0.05). However, the juiciness of the breast meat samples analysed on the 7th day of storage showed significant improvement. The intensities of meat juiciness between C and HSA0.7, and HS0.7 and HSA0.7 significantly differed at a significance level of *p* < 0.05 and *p* < 0.01, respectively. Additionally, a previously published study did not observe significant differences pertaining to juiciness when broilers were fed with HS [2]. It can be concluded that the application of HS in broiler diet does not significantly affect overall sensory quality, but it can improve some attributes like juiciness. However, the precise underlying mechanism of the effect of HS on the sensory characteristics of breast meat is unknown and requires further research.

The MFA statistical method indicated significant differences in analysed qualitative parameters of breast meat samples. The main interest of this applied method was in the observations of statistically significant parameters, which were positively or negatively correlated at a level of significance (*p* < 0.05). The statistical analysis extracted the most significant variables with a minimum loss of information. The total variation of 58% was explained by the first two dimensions. The analogy of the control and both experimental groups of samples in their quality was sorted according to the main studied factors (experimental diet and storage). In the first two dimensions, according to experimental diet factor, a dissimilarity between C, HS0.7, and HSA0.7 was observed. These experimental groups were not plotted close together in the visualised graph of individuals (Figure 2). It can be concluded that breast meat from each group had different characteristics based on the analysed parameters. On the other hand, the groups of breast meat samples analysed on the 1st and 7th day of storage showed a high similarity.

## 5. Conclusions

From the results that have been carried out, we can conclude that 0.7% supplementation of HS in natural, as well as acidified form to broilers’ feed significantly affected the composition and quality of breast meat. The content of meat fat and pH decreased and meat had a lighter colour. We also recorded a significant impact of HS feed addition on meat quality during storage. The oxidative stability and sensory variables of meat were better when compared to the control. When evaluating the natural and acidified form of HS on the quality of breast muscle meat, we observed a comparable effect. The improved effect of the acidified form of HS on growth parameters or meat quality was not confirmed. The addition of 0.7% natural HS represents a good potential for a significant increase in the quality of the meat produced, as well as for a potential improvement in the growth parameters of the poultry. However, the revealing of the detailed mechanism of HS action requires further research.

## Figures and Tables

**Table 1:** The composition of humic substances (HS) supplements.

Components	HS	HSA
Humic acids in dry matter, %	min. 65.00	min. 60.00
Free humic acids in dry matter, %	min. 60.00	min. 50.00
Fulvic acids, %	min. 5.00	min. 5.00
Formates, mg/kg	-	32,400
Calcium, mg/kg	42,278	51,100
Sodium, mg/kg	7111	6818
Magnesium, mg/kg	5111	4855
Potassium, mg/kg	903	874
Ferrum, mg/kg	19,046	18,094
Cuprum, mg/kg	15.00	14.25
Zinc, mg/kg	37.00	35.15
Manganese, mg/kg	142.00	135
Cobalt, mg/kg	1.24	1.18
Selenium, mg/kg	1.67	1.59
Vanadium, mg/kg	42.10	40.00
Molybdenum, mg/kg	2.70	2.57
Crude fibre, g/kg	24.3	22.4
Particle size, μm	<100	<100
Humidity, %	max. 21	max. 15
pH	5.8	5.4

HS: dietary natural humic substances; HSA: acidified humic substances with formic acid.

**Table 2:** The content of nutrients in final diet (BR3).

Components	C	HS0.7	HSA0.7
Corn grain, %	44	43.7	43.7
Wheat grain, %	26.2	25.8	25.8
Soybean meal, %	20	20	20
Dehulled sunflower meal, %	4	4	4
Rapeseed oil, %	0	0	0
Lard, %	2	2	2
Limestone, %	0.95	0.95	0.95
Monocalcium phosphate, %	1.6	1.6	1.6
Salt, %	0.25	0.25	0.25
Amino acids, vitamins, trace elements, %	1	1	1
Dietary natural humic substances, %	-	0.7	-
Acidified humic substances, %	-	-	0.7
Dry matter, g/kg	1000.00	1000.00	1000.00
Crude protein, g/kg	208.07	207.70	207.91
Crude fat, g/kg	52.20	51.20	51.77
Crude fibre, g/kg	45.05	45.90	46.60
Starch, g/kg	497.52	487.53	487.26
Ca, g/kg	7.76	8.70	8.80
P, g/kg	5.15	5.18	5.53
Metabolisable energy, MJ/kg	13.87	13.66	13.68

C: control group; HS0.7: broilers’ diet supplemented with 0.7% of dietary natural HS; HSA0.7: broilers’ diet supplemented with 0.7% of HS acidified with formic acid.

**Table 3:** The results of the growth performance and carcass yield of broilers.

Parameters	C	HS0.7	HSA0.7	Pooled SEM	*p* Value
Final weight, g	2319.00	2395.50	2387.07	25.29	0.142
Total feed consumption, g	3658.57	3696.03	3726.17	73.27	0.814
Total weight gain, g	2272.10	2348.83	2338.03	25.21	0.145
Feed conversion	1.61	1.57	1.59	0.02	0.317
Carcass yield, %	73.81	75.00	76.56	0.82	0.069
Breast without bone, %	30.51	31.97	30.53	0.91	0.440
Thighs with bone, %	28.34	28.17	29.07	0.66	0.611
Wings, %	10.24 ^a^	8.85 ^b^	8.90 ^b^	0.26	0.001
Hull, %	26.52	25.64	24.92	0.75	0.321

C: control group; HS0.7: broilers’ diet supplemented with 0.7% of dietary natural HS; HSA0.7: broilers’ diet supplemented with 0.7% of HS acidified with formic acid; SEM: standard error of the mean; ^a, b^ Means not sharing the same superscripts in row are significantly different (Tukey’s post hoc test, *p* < 0.05).

**Table 4:** The results of the physicochemical analyses of breast meat samples.

Physicochemical Parameters	C	HS0.7	HSA0.7	Pooled SEM	*p* Value
Dry matter, %	25.46 ^a^	25.31 ^a^	24.39 ^b^	0.20	0.017
Water content, %	74.54 ^a^	74.69 ^a^	75.61 ^b^	0.20	0.017
Fat, %	2.94 ^a^	2.28 ^b^	2.53 ^b^	0.08	0.003
Total protein, %	21.48 ^a^	22.03 ^a^	20.76 ^b^	0.17	0.001

C: control group; HS0.7: broilers’ diet supplemented with 0.7% of dietary natural HS; HSA0.7: broilers’ diet supplemented with 0.7% of HS acidified with formic acid; SEM: standard error of the mean; ^a,b^ Means not sharing the same superscripts in row are significantly different (Tukey’s post hoc test, *p* < 0.05).

**Table 5:** The results of pH and malondialdehyde (MDA) content determination in breast meat samples during storage (mg/kg).

Parameter	Storage	C	HS0.7	HSA0.7	Pooled SEM	*p* Value		
						D × S	D	S
pH	1st day	5.96 ^a^	5.80 ^b^	5.85 ^Ab^	0.02	0.020	<0.001	0.022
	7th day	5.96 ^a^	5.79 ^b^	5.75 ^Bb^	0.02			
MDA, mg/kg	1st day	0.23 ^B^	0.20 ^B^	0.21	0.02	0.125	0.013	<0.001
	7th day	0.34 ^Aa^	0.28 ^Aab^	0.24 ^b^	0.02			
AA, %	1st day	42.44 ^Ab^	46.20 ^Aa^	45.84 ^Aa^	0.51	0.432	<0.001	<0.001
	7th day	31.89 ^Bb^	35.73 ^Ba^	36.48 ^Ba^	0.49			

MDA: malondialdehyde; AA: antioxidant activity; C: control group; HS0.7: broilers’ diet supplemented with 0.7% of dietary natural HS; HSA0.7: broilers’ diet supplemented with 0.7% of HS acidified with formic acid; SEM: standard error of the mean; D: main factor of broiler diet modification; S: main factor of samples storage for 7 days; ^a,b^ Means not sharing the same superscripts in row are significantly different (Tukey’s post hoc test, *p* < 0.05); ^A,B^ Means not sharing the same superscripts in a column are significantly different (Sidak’s post hoc test, *p* < 0.05).

**Table 6:** The results of instrumental colorimetric analysis of breast meat samples during storage.

Colorimetric Parameters	Storage	C	HS0.7	HSA0.7	Pooled SEM	*p* Value		
						D × S	D	S
L*	1st day	56.49 ^b^	58.05 ^Bab^	58.12 ^Ba^	0.57	0.474	<0.001	<0.001
(lightness)	7th day	57.76 ^b^	59.97 ^Aa^	60.54 ^Aa^	0.34			
a*	1st day	16.70 ^a^	14.56 ^b^	14.37 ^b^	0.35	0.872	<0.001	0.081
(redness)	7th day	17.09 ^a^	15.22 ^b^	14.71 ^b^	0.29			
b*	1st day	10.91 ^A^	10.77	11.13	0.59	0.283	0.152	0.007
(yellowness)	7th day	8.77 ^Bb^	9.84 ^ab^	10.63 ^a^	0.47			
C*	1st day	20.12 ^a^	18.34 ^b^	18.22 ^b^	0.36	0.408	<0.001	0.271
(chroma)	7th day	19.28	18.24	18.23	0.33			
h	1st day	33.15 ^A^	35.70	37.81	1.80	0.457	<0.001	0.005
(hue angle)	7th day	27.22 ^Bb^	32.23 ^ab^	35.91 ^a^	1.41			

C: control group; HS0.7: broilers’ diet supplemented with 0.7% of dietary natural HS; HSA0.7: broilers’ diet supplemented with 0.7% of HS acidified with formic acid; D: main factor of broiler diet modification; S: main factor of samples storage for 7 days; ^a−b^ Means not sharing the same superscripts in row are significantly different (Tukey’s post hoc test, *p* < 0.05); ^A−B^ Means not sharing the same superscripts in a column are significantly different (Sidak’s post hoc test, *p* < 0.05).
